# Spin‐Torque Memristors Based on Perpendicular Magnetic Tunnel Junctions for Neuromorphic Computing

**DOI:** 10.1002/advs.202004645

**Published:** 2021-03-08

**Authors:** Xueying Zhang, Wenlong Cai, Mengxing Wang, Biao Pan, Kaihua Cao, Maosen Guo, Tianrui Zhang, Houyi Cheng, Shaoxin Li, Daoqian Zhu, Lin Wang, Fazhan Shi, Jiangfeng Du, Weisheng Zhao

**Affiliations:** ^1^ Fert Beijing Institute MIIT Key Laboratory of Spintronics School of Integrated Circuit Science and Engineering Beihang University Beijing 100191 China; ^2^ Beihang‐Goertek Joint Microelectronics Institute Qingdao Research Institute of Beihang University Qingdao 266000 China; ^3^ Truth Instruments Co. Ltd. Qingdao 266000 China; ^4^ CAS Key Laboratory of Microscale Magnetic Resonance and Department of Modern Physics University of Science and Technology of China Hefei 230026 China

**Keywords:** chiral spin vortices, magnetic tunnel junctions, memristors, neuromorphic computing, spintronics

## Abstract

Spin‐torque memristors are proposed in 2009, and can provide fast, low‐power, and infinite memristive behavior for neuromorphic computing and large‐density non‐volatile memory. However, the strict requirements of combining high magnetoresistance, stable domain wall pinning and current‐induced switching in a single device pose difficulties in physical implementation. Here, a nanoscale spin‐torque memristor based on a perpendicular‐anisotropy magnetic tunnel junction with a CoFeB/W/CoFeB composite free layer structure is experimentally demonstrated. Its tunneling magnetoresistance is higher than 200%, and memristive behavior can be realized by spin‐transfer torque switching. Memristive states are retained by strong domain wall pinning effects in the free layer. Experiments and simulations suggest that nanoscale vertical chiral spin textures can form around clusters of W atoms under the combined effect of opposite Dzyaloshinskii–Moriya interactions and the Ruderman–Kittel–Kasuya–Yosida interaction between the two CoFeB free layers. Energy fluctuation caused by these textures may be the main reason for the strong pinning effect. With the experimentally demonstrated memristive behavior and spike‐timing‐dependent plasticity, a spiking neural network to perform handwritten pattern recognition in an unsupervised manner is simulated. Due to advantages such as long endurance and high speed, the spin‐torque memristors are competitive in the future applications for neuromorphic computing.

## Graphical abstract

Nanoscale spin memristors are realized based on magnetic tunnel junctions with large tunneling magnetoresistance ratio. Strong domain wall pinning effects in the free layer with an atom‐thick tungsten insertion is shown to be the origin of the memristive behavior. Spike‐timing‐dependent plasticity of the device is studied. A spiking neural network is built, and handwritten pattern recognition is numerically demonstrated.

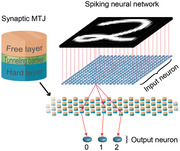

## 1 Introduction

Memristors are considered to be essential elements for realizing neuromorphic computing.^[^ ^1^, ^2^, ^3^ ^]^ Traditional memristors rely on ion motion and ionic valence changes in materials.^[^ ^1^, ^4^, ^5^ ^]^ However, most of them suffer from certain limitations, such as finite endurance^[^ ^6^ ^]^ or relatively low switching speed,^[^ ^1^ ^]^ which hinder their real applications in systems requiring long endurance such as neural networks with “on‐chip” learning. Spintronic devices, in which the state is modulated by magnetic variation and thus promise a much longer endurance, provide an alternative solution.^[^ ^7^, ^8^ ^]^ The concept of a spin‐torque memristor based on the current‐induced magnetic domain wall motion in the free layer of a magnetic tunnel junction (MTJ) was first proposed in 2009.^[^ ^9^ ^]^ Nevertheless, a real device with nanoscale dimension and all‐spin‐torque operation, for example, without the assistance of an external magnetic field, is still missing. The intermediate tunneling magnetoresistance (TMR) is difficult to stabilize against thermal activation or stimulating currents, especially in devices with nanoscale dimensions.^[^ ^10^ ^]^ A free layer in the partially switched state with domain walls usually has higher energy than monodomain states because both Heisenberg exchanges and magnetic anisotropy favor a collinear spin texture. Several possible solutions have been proposed, such as creating an intermediate state with the assistance of shape anisotropy,^[^ ^11^ ^]^ manipulating memristive switching through domain wall pinning in some complex geometries,^[^ ^12^, ^13^ ^]^ or by engineering the reference layer.^[^ ^14^ ^]^ However, these solutions require a large device size or an external magnetic field to realize memristive behaviors. The interfacial Dzyaloshinskii–Moriya interaction (DMI),^[^ ^15^, ^16^ ^]^ a form of antisymmetric exchange that favors a chiral spin texture, makes it possible to obtain intrinsically stable noncollinear magnetic structures in nanometer‐scale magnet and provides new possibilities to realize memristive MTJs with nanoscale dimensions.^[^ ^17^, ^18^ ^]^

In this work, we experimentally demonstrate a nanoscale spin‐torque memristor based on a perpendicular‐anisotropy MTJ with W‐inserted free layer. A high TMR ratio, a low resistance‐area product (RA), and spin‐polarized‐current‐induced switching are achieved. The memristive behavior is proved to originate from strong domain wall pinning effects in the free layer. Measurements with nitrogen‐vacancy (NV) color center in diamond indicate that the saturated magnetization is quite homogeneous in the free layer of MTJ stacks. Whereas, experiments and simulations suggest that a type of vertical chiral spin vortex could form around clusters of W atoms under the combined effect of Ruderman–Kittel–Kasuya–Yosida (RKKY) interactions and interfacial DMIs.^[^ ^15^, ^16^ ^]^ This spin texture leads to the fluctuations of the domain wall surface energy, which may be one reason for the strong domain wall pinning effects. The spike‐timing‐dependent plasticity (STDP) functionality is experimentally validated. A compact model of the spin‐torque memristors is created according to experimental data and is integrated into a spiking neural network (SNN), which was then employed for the Mixed National Institute of Standards and Technology database (MINST) handwritten pattern recognition.^[^ ^19^ ^]^ Arabic handwritten numeral can be recognized with our devices. Thanks to advantages such as long endurance,^[^ ^7^, ^20^, ^21^ ^]^ the spin‐torque memristor studied in this work provides a new opportunity for the application of spintronic devices in neuromorphic computing with “on‐chip” learning.

## 2 Results and Discussion

### 2.1 Memristive MTJs

**Figure 1:**
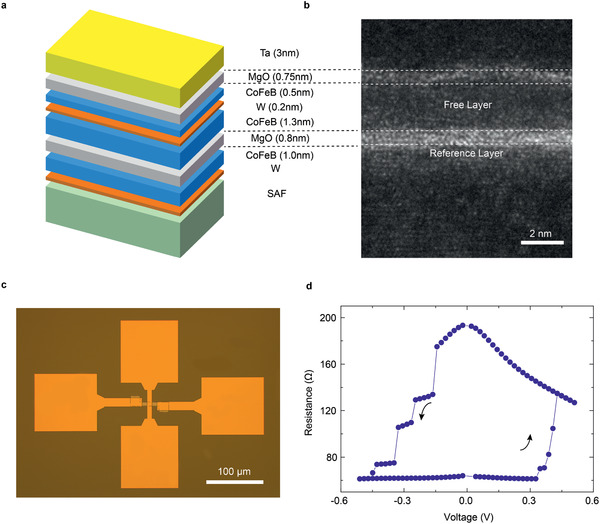
Layer structure and electric test of the device. a) The stack structure of the MTJ. b) Cross‐sectional TEM image showing the layer quality of the stack. c) Optical microscopy image of an MTJ with four electrodes. d) STT‐induced switching using stepwise increasing voltage, with each step lasting 100 ms. A 20‐mT perpendicular field is applied during the test to compensate the bias of stray fields from the reference layer, the same is also true throughout the text.

**Figure** **1**a introduces the layer structure used to fabricate the memristive MTJ device: synthetic antiferromagnet/W (0.25)/CoFeB (1.0)/MgO (0.8)/CoFeB (1.3)/W (0.2)/CoFeB (0.5)/MgO (0.75)/Ta (3.0) (thickness in nanometer). The stack is prepared with a Singulus magnetron sputtering machine and is annealed at 390 °C for 1 h after deposition. In contrast to traditional MTJs with a single‐free layer structure, in this study, an atomic‐thickness W layer is inserted between two free layers during sputtering deposition to engineer the free layer properties.^[^ ^22^, ^23^ ^]^ A transmission electron microscopy (TEM) image of the multilayer stack is presented in Figure 1b.

The MTJ film with composite free layer is patterned into circular nanopillars with a 200‐nm radius (R) using electron beam (e‐beam) lithography and Ar ion milling and is instrumented with gold electrodes, as shown in Figure 1c. Then, continuous and stepwise increasing voltage is applied and resistance of the device is measured simultaneously. As shown in Figure 1d, the spin‐transfer torque (STT)‐induced magnetization switching is achieved, with multiple intermediate states on both switching directions. A TMR ratio as large as 200% and an RA of 7 Ω µm^2^ are obtained at room temperature. The switching current is on the order of MA cm^−2^.

**Figure 2:**
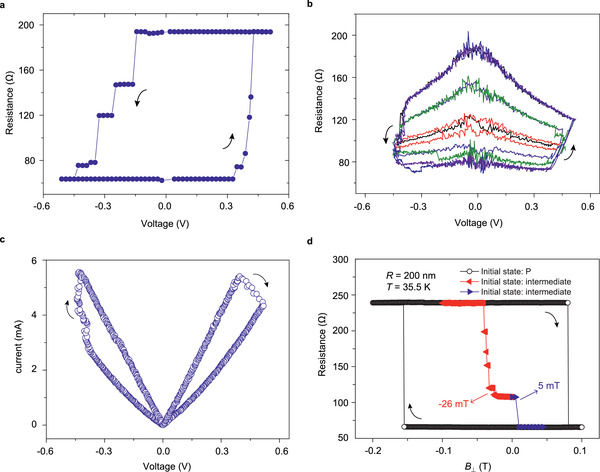
Spin‐torque‐induced and magnetic‐field‐induced switching of an MTJ with R = 200 nm. a) Resistance–voltage loop. In this measurement, a train of voltage pulses with a duration *T*_P_ of 100 ms and an increase of 0.02 V per step is applied. The resistance is measured using a voltage of 0.01 V after each stimulus pulse. b) Resistance–voltage minor loops. In this measurement, short voltage pulses with *T*_P_= 200 ns are applied. c) Current–voltage loop obtained using a train of voltage pulses with *T*_P_= 200 ns. The resistance is measured when switching voltage is applied in both B and C. d) Black: full resistance‐perpendicular field hysteresis loop of the device; red and blue: stability of intermediate state against external magnetic fields at 35.5 K.

To investigate the stability of the intermediate states, voltage pulses with a duration *T*_P_= 100 ms are applied and after each pulse, the resistance is immediately measured with a 0.01‐V reading voltage, as shown in **Figure** **2**a. The voltage‐dependent decrease of the antiparallel resistance is avoided using this low reading voltage.^[^ ^24^ ^]^ Intermediate states are reproduced, which correspond well with those in Figure 1d, confirming their good stability. Furthermore, sequences of shorter voltage pulses with *T*_P_= 200 ns are applied to observe the STT‐switching and more intermediate states appeared, as shown in Figure 2b. As can be observed from the minor loops, the intermediate states are stable even when the polarity of the applied voltage is reversed. It is worth noting that the pulse width in our tests cannot be further compressed because of the oscillating noise. Much more intensive intermediate states can be expected if the stimulating pulse width scales down to nanoseconds, which is the typical spin‐flip time in STT‐induced switching. Figure 2c gives the current–voltage loop obtained by a train of *T*_P_= 200 ns switching voltage pulses. One can find that once the applied voltage increases over a threshold value, the resistance of the device varies quasi‐continuously, which is typical memristive behaviors.

The stability of the intermediate states against external magnetic fields is checked. First, the resistance‐magnetic field hysteresis loop in a device with R = 200 nm is measured at a low temperature, as shown in Figure 2d. As expected, the coercive field is very large in such a small device and no intermediate state is observed. Whereas, after creating an intermediate state with a STT current and then applying an external field, we find that the intermediate state remained stable under certain external magnetic fields. The field needed to destroy the intermediate state is ≈15.5 mT after offsetting the bias caused by the stray field from the reference layer.

### 2.2 Strong Domain Wall Pinning Effect in the Composite Free Layer

The magnetoresistance of an MTJ depends on the relative magnetic state of the free layer with respect to the pinned layer.^[^ ^24^ ^]^ A stable intermediate state is rare in traditional single‐free layer MTJs with submicron dimensions since once magnetic switching begins, the nucleated domain wall moves forward immediately and leads to the complete switching.^[^ ^10^ ^]^ Here, we get a different process in the composite free layer MTJs.

**Figure 3:**
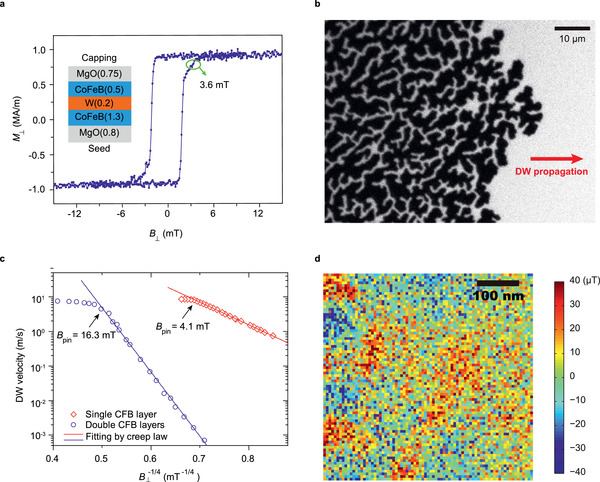
Strong domain wall pinning in a MgO/CoFeB/W/CoFeB/MgO film (FL‐film). a) The perpendicular hysteresis loop of the FL‐film. b) Kerr image showing the dendritic trace of the domains after domain wall motion driven by a perpendicular field of 3.6 mT in the FL‐film. c) Velocities of domain wall motion driven by a perpendicular field in FL‐film (in blue circle) and a W/CoFeB (1.0 nm)/MgO film (in red diamond), and the linear fit using the creep law. d) Scanning image of the stray fields distribution above a saturated FL‐film. The NV center is 20.7±6.7 nm above the sample and the quantization axis tilts 35° from the sample plane.

To obtain a deeper insight into the properties of the composite free layer, we deposited a MgO/CoFeB/W/CoFeB/MgO film with the same structure as that in the MTJ stack (called FL‐film in the following). The deposition and annealing conditions remained the same. As seen from the hysteresis loop of this film (see **Figure** **3**a) and the MTJ stack (see the Supporting Information 1 (online)), the two CoFeB layers are globally ferromagnetically coupled and show strong perpendicular magnetic anisotropy. An intermediate state of the MTJ should not be caused by inconsistent magnetization of the upper free layer (Up‐layer) and the lower free layer (Lw‐layer). The field‐induced magnetization reversal of the free layers appears to be gradual, indicating that the threshold field for domain wall propagation is much larger than the domain wall nucleation field in this film. Next, the field‐induced domain wall motion in the FL‐film is imaged using Kerr microscopy. Figure 3b shows a dendritic trace after domain wall motion induced by a magnetic field of 3.6 mT, which appears much rougher than an ordinary single‐layer CoFeB film with low pinning effect,^[^ ^25^ ^]^ indicating a strong pinning effect in the composite free layer film. The velocity of the field‐induced domain wall motion is measured and it leaves the thermally activated creep regime^[^ ^26^ ^]^ until the driving field reaches ≈*μ*_0_H_C_ = 16 mT, as shown in Figure 3c. This critical field (conventionally called intrinsic pinning field) can be seen as an indicator of the domain wall pinning strength in a magnetic material, below which the wall motion is not possible without the assistance of thermal activation.^[^ ^26^ ^]^ This value is consistent with the field (15.5 mT, see Figure 2d) required to destroy an intermediate state in the MTJ device at low temperature, confirming that the intermediate states are stabilized by the strong domain wall pinning effect in the free layer.

According to Leon Chua's definition, a memristor can be described as, *v*  =  *R*(*w*, *i*)*i* and $\frac{dw}{dt}=\;f\left(w,i\right)$, where *v* and *i* are the voltage and current. *w* can be a set of state variables and *R* and *f* can in general be explicit functions of time.^[^ ^27^, ^28^ ^]^ Obviously, the behavior of the present device consists of this definition, where w can be defined as the domain wall position. Theoretically, the number of intermediate states depends on the pinning sites distribution. Increasing the density and strength of pinning sites allows to obtain more intermediate states in a device with finite size.

### 2.3 Hybrid Chiral Domain Wall Structure

The domain wall pinning effect in a heavy metal/CoFeB/MgO stack is usually very weak.^[^ ^25^ ^]^ For example, we measured the domain wall motion velocity in a W/CoFeB (1 nm)/MgO film for comparison and found the intrinsic pinning field is 4.1 mT, as shown in Figure 3c. The domain wall pinning effects in double layer film with W insertion is four times larger than CoFeB single‐layer films.

Scanning microscopy with NV center in diamond is used to check the magnetic quality of the FL‐film.^[^ ^29^, ^30^ ^]^ The NV center is placed 20.7±6.7 nm above a magnetically saturated FL‐film and the distribution of stray fields along the NV center quantization axis is obtained, as shown in Figure 3d. Supposing a fluctuation of the magnetization by 10% occurs in a 10 nm × 10 nm local area, which is a minimum dimension for an eventual defect to pin a domain wall, the fluctuation of the stray field caused by this defect should be in the order of 300 μT according to simulations (see Supporting Information 1 (online)). This fluctuation may be caused by a local change of the magnetic layers thickness or the saturation magnetization. Obviously, the measurement results via NV center rule out the major role of this kind of pinning effect.

**Figure 4:**
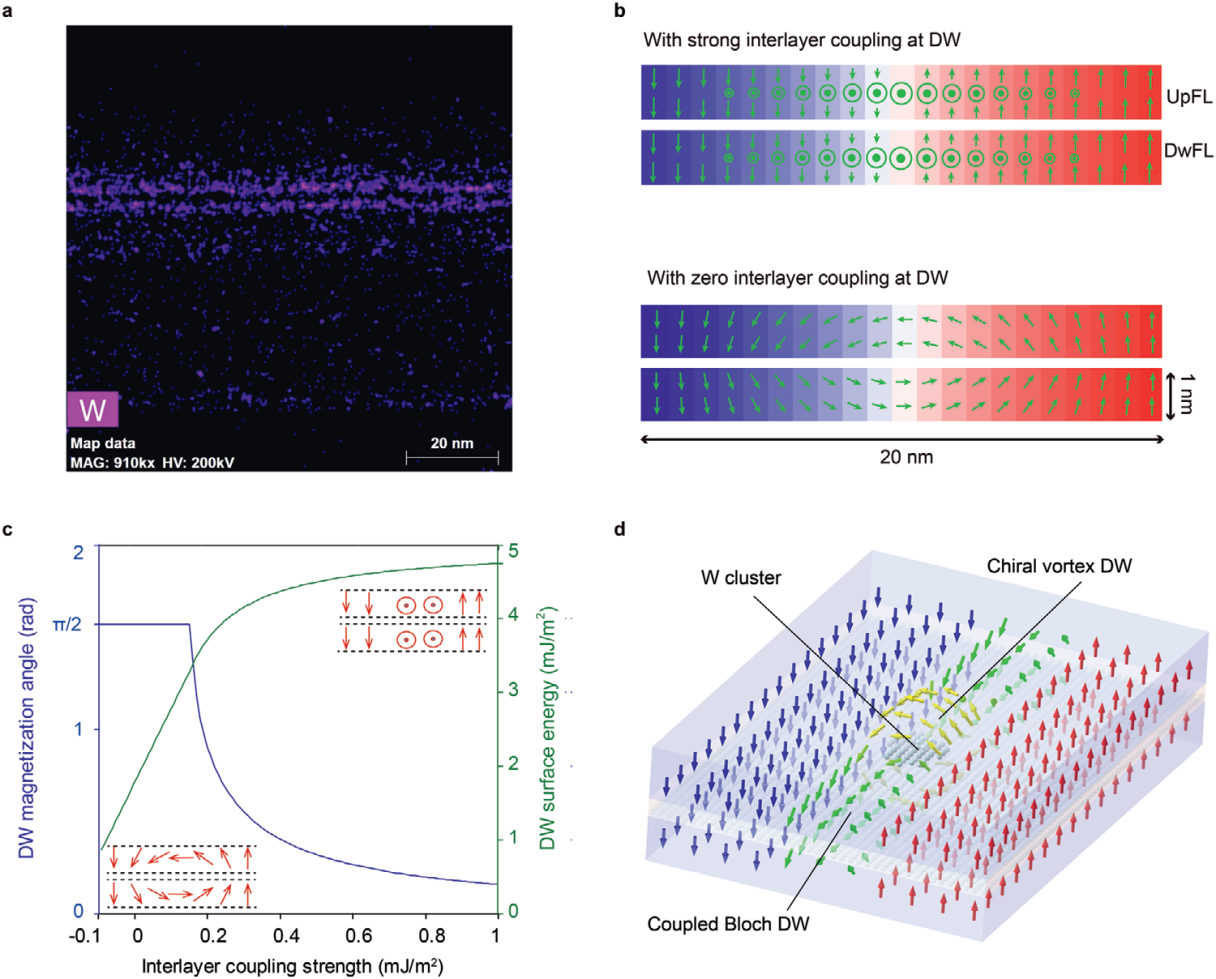
Domain wall profile in CoFeB/W/CoFeB multilayers dominated by the competition between interlayer coupling and opposing DMIs. a) EDS mapping showing the inhomogeneous distribution of W atoms in the multilayer stack. b) Side‐view of domain wall profiles under different interlayer coupling strengths obtained via micromagnetic simulations. c) Variations in the azimuth angle of the domain wall magnetization (0 means chiral vortex wall and *π*/2 means coupled Bloch wall) and the surface energy as functions of the interlayer coupling strength. d) Schematic showing the chiral vortex domain wall structure around a W cluster.

According to the energy‐dispersive X‐ray spectroscopy (EDS) mapping (**Figure** **4**a), the spatial distribution of W atoms in the composite free layer of MTJ stack is inhomogeneous, with some stochastic overlap and some breakage, which may be caused by atom diffusion during the annealing process. According to the RKKY theory, two ferromagnets separated by a thin metal layer exhibit periodic ferro‐/antiferromagnetic exchange, where the period depends on the type and lattice structure of the metal.^[^ ^31^ ^]^ Based on the experimental data reported by S. Parkin^[^ ^32^ ^]^ and our fitting results according to the RKKY law, two ferromagnets neighboring a W spacer begin to exhibit an antiferromagnetic coupling when the W‐thickness reaches ≈0.44 nm, reaching a peak at a W‐thickness of 0.55 nm, corresponding to approximately two atomic layers.^[^ ^33^ ^]^ The antiferromagnetic coupling between CoFeB layers separated by 0.6‐nm thick W is also observed in a recent study.^[^ ^34^ ^]^ Therefore, although the two free layers of our MTJ stack exhibit globally ferromagnetic exchange, the stochastic overlapping of the W atoms (W clusters) may result in antiferromagnetic exchange in some local regions.

On the other hand, considerable DMIs can arise at a W/CoFeB interface because of the spin‐orbit coupling,^[^ ^35^, ^36^ ^]^ and also at a CoFeB/MgO interface.^[^ ^37^, ^38^, ^39^ ^]^ This interaction is particularly large for a Fe‐rich CoFeB composition, as in our case, and promotes a chiral magnetic texture in the magnetic layer.^[^ ^40^, ^41^ ^]^ We have measured the DMI in films with different symmetry breaking via asymmetric domain wall motion when both the perpendicular field and in‐plane field are applied. The DMI is about 0.45 mJ m^−2^ and favors a left‐handed chirality in a MgO/CoFeB(1.7 nm)/W film; while DMI reaches 0.65 mJ m^−2^ and favors a right‐handed chirality in a W/CoFeB(1 nm)/MgO film (see Supporting Information 1 (online)). These results suggest that in the composite free layer of MTJs, opposite chiralities are favored for the domain walls in the Up‐layer and Lw‐layer. On the one hand, because of the global ferromagnetic coupling of the two free layers, the total DMI cancels out (only 0.02 mJ m^−2^ is detected in the FL‐film) and the domain wall configuration shows no chirality (see the Supporting Information 1 (online)). In contrast, when the exchange coupling between the Up‐layer and Lw‐layer is weak or even antiferromagnetic in a local region where W atoms overlap, the domain wall chiralities in the two free layers should be determined by the corresponding DMIs. The magnetization of the domain walls center in the two free layers are opposite, and a chiral vortex could form around the W cluster.

The magnetic state of the two 1‐nm‐thick ferromagnetic layers separated by a 0.2‐nm spacer is simulated using the OOMMF code,^[^ ^42^ ^]^ as shown in Figure 4b. In the first (second) case, the ferromagnetic coupling strength *J*_ex_ is set to be 1 mJ m^−2^ (0.01 mJ m^−2^), where a positive (negative) sign of *J*_ex_ means ferromagnetic (antiferromagnetic) coupling. The interfacial DMI constant D is set to 0.5 mJ m^−2^ in both cases, with a negative (positive) sign in the Up‐layer (Lw‐layer). The results confirm the above analysis: a coupled Bloch domain wall forms in the two free layers with strong coupling, while a vertical vortex forms under weak coupling, the chirality of which is dominated by the DMIs in each layer.

Furthermore, the magnetization direction in the center of a domain wall and the domain wall surface energy as a function of the interlayer coupling strength are calculated and given in Figure 4c (see detailed calculations in Supporting Information 1(online)). As the interlayer coupling strength decreases below 0.35 mJ m^−2^, the domain wall profile transforms to a chiral vortex, and the domain wall surface energy drops rapidly. In fact, the formation of the chiral vortex domain wall minifies both the energy associated with DMIs and with dipole–dipole interactions.^[^ ^43^ ^]^ According to the fitting result based on the data reported by S. Parkin,^[^ ^32^ ^]^ the interlayer coupling decreases from 0.6 mJ m^−2^ to −0.03 mJ m^−2^ as the thickness of the W increases from 0.2 to 0.55 nm in ferromagnet/W/ferromagnet structure. When a moving domain wall encounters a W cluster, the domain wall will locally transform into a chiral vortex, as illustrated in Figure 4d. The energy well due to the transition of domain wall configuration could be one important reason for the strong pinning effects in the free layer of our MTJ stack (see the video showing this effect in Supporting Information 2 (online)). Indeed, the transition of magnetic chirality from a vortex‐Néel domain wall to a degenerate Bloch–Néel wall mediated by increasing RKKY interactions has been experimentally observed in similar structures.^[^ ^44^ ^]^ It should be noted that, according to our model, even a partial rotation of the domain wall angle could cause a large fluctuation of wall energy (Figure 4c). Besides, the local variation of magnetic anisotropy due to the inhomogeneous distribution of W or local change of exchange coupling due to W—Co or W—Fe alloying may also contribute to the domain wall pinning effect.

Moreover, the dendritic domains morphology after field‐driven domain wall motion (Figure 3b) also suggest that the strong demagnetizing field due to the thick composite free layers in this sample (1.8 nm in total) plays a non‐negligible role in maintaining the uncompleted switching state in the free layers (see the Supporting Information 1 (online)).

### 2.4 Spike‐Timing‐Dependent Plasticity

**Figure 5:**
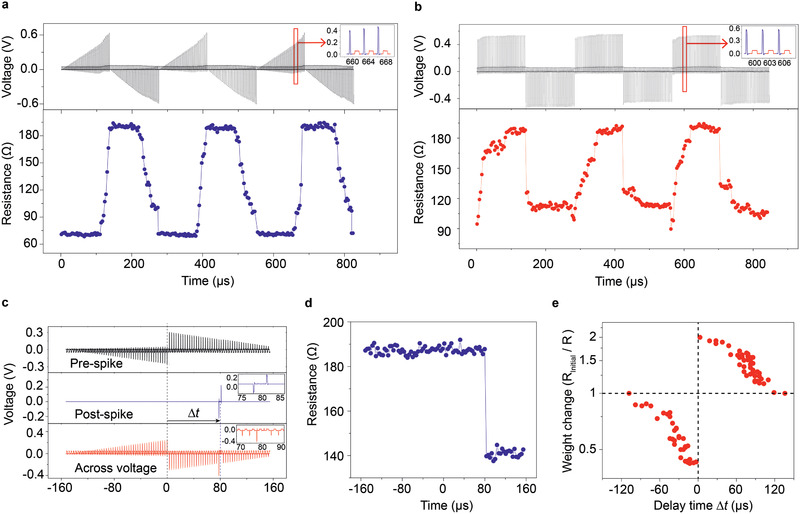
a) Plasticity explored by pulse sequence with ramped amplitude (from 0 to 6.2 V/−5.5 V with 0.01‐V increase per step) and constant duration (*T*_P_ = 200 ns per pulse). The upper plot shows the stimulating and detecting pulses (blue and red in the inset, respectively). A pulse with 0.05‐V amplitude and 1‐µs duration is applied after each stimulating pulse as the detecting signal. The lower plot shows the corresponding detected resistance. b) Plasticity explored by constant amplitude pulses sequence (0.54 V/−0.44 V) with *T*_P_ = 200 ns. The resistance is measured via low‐voltage pulse (0.05 V, *T*_P_ = 1 µs). c) The pre‐spike and post‐spike waveforms and the across voltage obtained as the superposition of the pre‐ and post‐spikes. d) An example of the resistance change induced by an across voltage with Δ*t* = 80 µs. e) STDP learning curve.

Due to the memristive and non‐volatile magnetoresistance, this two‐terminal MTJ can be used as synapse for neuromorphic computing.^[^ ^45^ ^]^ The plasticity, an essential property of an electronic synapse,^[^ ^46^ ^]^ is investigated by applying two types of spike stimulus. First, a train of spike voltage pulses with stepwise increasing magnitude is applied and the resistance is measured immediately after each stimulating pulse, as shown in the top panel and inset of **Figure** **5**a. When the spike voltage reaches a threshold value (0.39 V/−0.37 V), the resistance of the device begins to increase/decrease gradually, corresponding to the potentiation/depression function of the synapse. The non‐volatile quasi‐continuous variation of resistance with stimulus shown here is a characteristic of long‐term functional synaptic plasticity.^[^ ^2^ ^]^ In the second case, the plasticity is explored using sequences of voltage pulses with constant amplitude (0.54 V/−0.44 V) and duration (*T*_P_ = 200 ns), as shown in Figure 5b, and plastic behavior is also observed. It is worth noting that with different types of stimulating signals, two different memristive profiles are obtained on the same device and both of them could be employed in neuromorphic computing.^[^ ^2^, ^47^ ^]^

Then, STDP, a fundamental learning function for artificial neural networks,^[^ ^47^, ^48^ ^]^ is investigated based on these devices. Two sequences of pulses ( *T*_P_= 200 ns) with opposite polarity, the amplitude of which ramped below the threshold switching voltage, are used as pre‐spike. A couple of opposite‐voltage pulses (*T*_P_ = 200 ns and ±0.2 V), with a delay time Δ*t* from the center of pre‐spike, are used as post‐spike, as shown in Figure 5c. Here, Δ*t* is defined as positive (negative) when the pre‐spike stimulus is applied before (after) the post‐spike. Since the amplitude of both the pre‐ and post‐spike stimulus are below the switching voltage of the device, the resistance variation is determined only by the polarity and amplitude of the pick of the across voltage, which depends uniquely on the delay time Δ*t*. Figure 5d gives an example showing the resistance variation corresponding to an across voltage with Δ*t* = 80 µs. We define the synaptic weight as the ratio of the initial resistance to the resistance after spiking. Then, we perform a set of tests to observe the weight changes under varying delay time Δ*t*, ranging from −110 to 140 µs, and the results are given in Figure 5e. Statistically, when the pre‐spike occurs before (Δ*t*>0) the post‐spike, the synaptic weight increases, mimicking the potentiation of synapse in a neural system, vice versa; The smaller |Δ*t*|, meaning the higher correlation between the two spikes, the deeper potentiation/depression of synapse.

### 2.5 Spin Memristor‐Based SNNs

**Figure 6:**
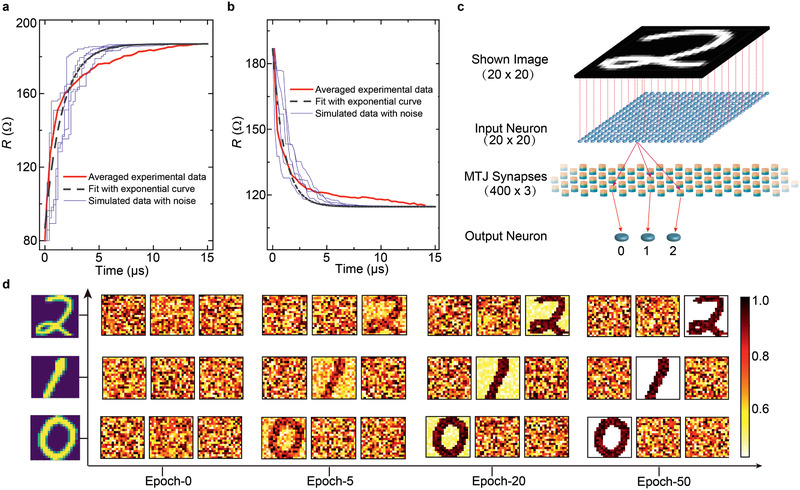
Spin‐torque memristor‐based SNN implementation and simulations. Resistance change in spin‐torque memristor corresponding to the a) P‐AP (V = 0.54 V) and b) AP‐P (V = −0.44 V) switching process. Red lines represent the averaged result based on 10 experimental tests and the dashed black lines represent the fitting result with the exponential function. Thin blue lines show the switching behavior given by the built model (with noise) used in simulations for 5 tests under identic stimulating voltage. c) The topological structure of proposed SNN with 20 × 20 pre‐neurons connected to 3 post‐neurons through a 400 × 3 artificial synapses array. Each pixel of the images shown to the network is associated with 3 handwritten numerals (0, 1, and 2). d) Synaptic weight evolution of the MTJs matrix in the SNN during unsupervised learning session for 50 epochs, trained with the three numerals. The color bar on the right indicates the conductance range from 1/*R*_ap_ to1/*R*_p_.

In order to investigate the availability and performance of the device in spiking neuromorphic networks, a device‐system simulation with a hierarchical framework is performed. A behavioral model of the device that matches the experimental data is developed and then integrated as synapse in the network, interfacing with the Leaky‐Integrate‐Fire neurons. For both the AP‐P and P‐AP switching, the variation of the resistance along with the stimulating time can be phenomenologically fitted with an exponential function, with a characteristic time constant *τ* for each given voltage, as shown in **Figure** **6**a,b. ln *τ* is observed to be linear to the voltage, consistent with the domain wall pinning‐dominated switching model.^[^ ^49^ ^]^ A noise factor is added into the device model considering the device's variations and stochastic nature of the domain wall depinning effect. Some examples of the switching behaviors of the device model are given in Figure 6a,b. Then, unsupervised learning is conducted on a subset of the MNIST database^[^ ^19^ ^]^ as shown in Figure 6c. Three stylized Arabic numerals (0, 1, and 2) are used for both training and testing. To classify the 20 × 20‐pixel black‐and‐white images into 3 classes, a two‐layer network with 20 × 20 neurons as input layer and 3 neurons as the output layer is built. Each input neuron is connected with one pixel of the image, which is then fed to the output layer through the synaptic connection built by spin‐torque memristors. Thus, the firing rates of output neurons were mainly determined by the plasticity of the spin‐torque memristor. The specified unsupervised learning protocol can be referred to.^[^ ^47^ ^]^ Figure 6d plots the synaptic conductance of spin‐torque memristors (or weights) learned by the system during the complete training session for all the letters. Initially, all the weights are randomly distributed between 1/*R*_ap_ to1/*R*_p_. Increasing the number of epochs from 0 to 5, 20th, and 50, the weight of the depressed synapses (purple lines) gradually converge to the lowest conductance and the potentiated synapses (yellow lines) converge to the highest conductance. After 50th epochs of unsupervised training, the neural network is trained and the letters are recognizable in the synaptic layer. The specified system simulation method and intermediate data could be found in the Supplementary Materials.

The resistance of the presented spin memristor is around hundreds of Ohms and the ON/OFF ratio is lower than conventional metal‐oxide memristors.^[^ ^28^ ^]^ These disadvantages could be overcome by using multiple devices in series as one synapse or implementing sense amplifiers in the synaptic array in real applications.^[^ ^50^ ^]^ Since the response speed of the device increases in an exponential manner with the applied voltage, a spiking time reduced to nanoseconds can be expected if the voltage is slightly increased. In fact, tens nanometers of STT‐induced domain wall motion are possible within this time scale (10). Considering a spiking voltage of about 0.6 V and the average device resistance of 130 Ω, the power consumption per spike is estimated to be in the order of 10 pJ for a single device. It is worth noting that the memristive behavior of our devices is achieved through magnetic switching, without ion motion or ionic valence changes. Their endurance is expected to be more than 10^15^ cycles,^[^ ^7^, ^20^, ^21^ ^]^ which is promising for neuromorphic computing that requires high reliability on devices, for example, neural networks with “on‐chip” learnings.^[^ ^51^ ^]^

## 3 Conclusion

In conclusion, a spin‐torque memristor with a high TMR ratio, a low RA, a low working current, and a nanoscale size is obtained by engineering an atomic‐thickness W spacer in the free layer of a MTJ. A memristive TMR is achieved during STT‐induced switching in both directions. By comparing the intrinsic pinning field for domain wall motion in the free layer films and the filed required to break the intermediate TMR at low temperature, it is proved that the memristive behavior of the device originates from strong domain wall pinning effects. NV center measurements prove that the magnetization of the free layer is quite homogenous in a saturated state. Experiments and micromagnetic simulations show that a chiral vortex domain wall could form at a cluster of W atoms because of the opposing interfacial DMIs and the RKKY interaction. The energy fluctuation induced by the domain wall structural transition between trivial coupled Bloch configuration and vertical chiral vortex configuration may be one reason for the strong pinning effect. The STDP functionality of the device has been experimentally demonstrated based on its synaptic property. A compact model is developed based on experimental data and system‐level simulation is performed, showing that our spin‐torque memristor can be performant and competitive for neuromorphic computing, such as unsupervised learning.

## 4 Experimental Section

##### Device Preparation

The MTJ films were deposited by a Singulus TIMARIS 200 mm magnetron sputtering machine at a base pressure of 3.75 × 10^−9^ Torr. Circular nanopillars were patterned by e‐beam lithography, followed by Ar ion milling and SiO_2_ insulation. After the lift‐off procedure, Ti/Au electrodes were evaporated for measurements.

##### Characterization of the Film

Fundamental properties of the MTJ stack and the FL stack, including the saturation magnetization and the perpendicular anisotropy, were both measured via VSM. Besides, the domain structure and domain wall motion in the FL film were observed using a Kerr microscope with a 400‐nm resolution. A fast‐perpendicular field with a rise time of sub‐microsecond, which is produced by a small magnetic coil, is used to measure the domain wall motion velocities in the magnetic film. The strength of DMIs in films with different structure asymmetries are measured via analyzing domain wall motion velocities when out of plane magnetic fields and in‐plane fields are applied simultaneously (see Supporting Information 1).

##### Electrical Measurements of the Device and Statistical Analysis

The setup for the electrical characterization of the spin‐torque memristor consists of a Lake Shore CRX‐VF cryogenic probe station, Keithley 4200, Keithley 6221 current sources, and 2182 nanovolt meters. The resistance of the device is measured using four‐probe method. The applied voltage and current are directly recorded to calculate the resistance of the device by the equation: R = V / I. Origin Software is used for data processing and analysis.

##### Micromagnetic Simulation

Micromagnetic simulations based on the OOMMF code were performed to observe the magnetic texture and domain wall pinning effect in the dual FLs. Simulations based on Mumax3 software were performed to quantify the effect of the demagnetizing field on the domain wall structure. Both simulations are based on solving the Landau–Lifshitz–Gilbert equation.

##### SNN Simulations

In order to simulate the implementation of SNN based on the proposed spin‐torque memristors device, a hybrid device‐system co‐simulation framework is utilized. A “top‐down” (algorithm) and “bottom‐up” (device modeling) simulation framework written by Python is developed to evaluate the performance of the STDP synaptic function based on the memristive behavior over a subset of standard MNIST handwritten dataset.

## Conflict of Interest

The authors declare no conflict of interest.

## Supporting information

Supporting Information

Supplemental Movie S1

## Data Availability

The data that support the findings of this study are available from the corresponding author upon reasonable request.

## References

[1] M. A. Zidan , J. P. Strachan , W. D. Lu , Nat. Electron. 2018, 1, 22.

[2] C. Wu , T. W. Kim , H. Y. Choi , D. B. Strukov , J. J. Yang , Nat. Commun. 2017, 8, 752. 28963546 10.1038/s41467-017-00803-1 PMC5622032

[3] T. Prodromakis , C. Toumazou , L. Chua , Nat. Mater. 2012, 11, 478. 22614504 10.1038/nmat3338

[4] S. Pi , C. Li , H. Jiang , W. Xia , H. Xin , J. J. Yang , Q. Xia , Nat. Nanotechnol. 2018, 14, 35. 30420759 10.1038/s41565-018-0302-0

[5] Z. Wang , S. Joshi , E. Savel'ev , H. J. Sergey , R. Midya , P. Lin , M. Hu , N. Ge , P. Strachan , Z. L. John , Q. Wu , M. Barnell , G.‐L. Li , L. Xin , R. S. W. Huolin , Q. Xia , J. J. Yang , Nat. Mater. 2016, 16, 101. 27669052 10.1038/nmat4756

[6] M. J. Lee , C. B. Lee , D. Lee , S. R. Lee , M. Chang , J. H. Hur , Y. B. Kim , C. J. Kim , D. H. Seo , S. Seo , U. I. Chung , I. K. Yoo , K. Kim , Nat. Mater. 2011, 10, 625. 21743450 10.1038/nmat3070

[7] J. J. Kan , C. Park , C. Ching , J. Ahn , Y. Xie , M. Pakala , S. H. Kang , IEEE Trans. Electron Devices 2017, 64, 3639.

[8] N. Locatelli , V. Cros , J. Grollier , Nat. Mater. 2013, 13, 11. 10.1038/nmat3823 24343514

[9] X. Wang , Y. Chen , H. Xi , H. Li , D. Dimitrov , IEEE Electron Device Lett. 2009, 30, 294.

[10] T. Devolder , J. Von Kim , F. Garcia‐Sanchez , J. Swerts , W. Kim , S. Couet , G. Kar , A. Furnemont , Phys. Rev. B 2016, 93, 024420.

[11] A. Chanthbouala , R. Matsumoto , J. Grollier , V. Cros , A. Anane , A. Fert , A. V. Khvalkovskiy , K. A. Zvezdin , K. Nishimura , Y. Nagamine , H. Maehara , K. Tsunekawa , A. Fukushima , S. Yuasa , Nat. Phys. 2011, 7, 626.

[12] J. Cai , B. Fang , C. Wang , Z. Zeng , Appl. Phys. Lett. 2017, 111, 182410.

[13] S. Lequeux , J. Sampaio , V. Cros , K. Yakushiji , A. Fukushima , R. Matsumoto , H. Kubota , S. Yuasa , J. Grollier , Sci. Rep. 2016, 6, 31510. 27539144 10.1038/srep31510 PMC4990964

[14] H. Zhong , Y. Wen , Y. Zhao , Q. Zhang , Q. Huang , Y. Chen , J. Cai , X. Zhang , R. Li , L. Bai , S. Kang , S. Yan , Y. Tian , Adv. Funct. Mater. 2018, 29, 1806460.

[15] A. R. Fert , Mater. Sci. Forum 1990, 59–60, 439.

[16] M. Bode , M. Heide , K. von Bergmann , P. Ferriani , S. Heinze , G. Bihlmayer , A. Kubetzka , O. Pietzsch , S. Blügel , R. Wiesendanger , Nature 2007, 447, 190. 17495922 10.1038/nature05802

[17] W. Legrand , J. Chauleau , D. Maccariello , N. Reyren , S. Collin , K. Bouzehouane , N. Jaouen , V. Cros , A. Fert , Sci. Adv. 2018, 4, eaat0415. 30035224 10.1126/sciadv.aat0415 PMC6054507

[18] X. Zhang , W. Cai , X. Zhang , Z. Wang , Z. Li , Y. Zhang , K. Cao , N. Lei , W. Kang , Y. Zhang , H. Yu , Y. Zhou , W. Zhao , ACS Appl. Mater. Interfaces 2018, 10, 16887. 29682962 10.1021/acsami.8b03812

[19] Y. Lecun , C. Cortes , C. J. Burges , “THE MNIST DATABASE of handwritten digits,” can be found under http://yann.lecun.com/exdb/mnist/, 2010.

[20] M. H. Kryder , C. S. Kim , IEEE Trans. Magn. 2009, 45, 3406.

[21] R. Carboni , S. Ambrogio , W. Chen , M. Siddik , J. Harms , A. Lyle , W. Kula , G. Sandhu , D. Ielmini , *Int. Electron Devices Meet. IEDM*, San Francisco 2017, p. 21.6.1.

[22] J. Y. Choi , D. G. Lee , J. U. Baek , J. G. Park , Sci. Rep. 2018, 8, 4.29311719

[23] S. Couet , J. Swerts , S. Mertens , T. Lin , Y. Tomczak , E. Liu , B. Douhard , S. Van Elshocht , A. Furnemont , G. S. Kar , IEEE Magn. Lett. 2016, 7, 1.

[24] J. C. Slonczewski , Phys. Rev. B 2005, 71, 024411.

[25] C. Burrowes , N. Vernier , J.‐P. Adam , L. Herrera Diez , K. Garcia , I. Barisic , G. Agnus , S. Eimer , J.‐V. Kim , T. Devolder , A. Lamperti , R. Mantovan , B. Ockert , E. E. Fullerton , D. Ravelosona , Appl. Phys. Lett. 2013, 103, 182401.

[26] R. Diaz Pardo , W. Savero Torres , A. B. Kolton , S. Bustingorry , V. Jeudy , Phys. Rev. B 2017, 95, 184434.

[27] L. Chua , M. K. Sun , Proc. IEEE 1976, 64, 209.

[28] D. B. Strukov , G. S. Snider , D. R. Stewart , R. S. Williams , Nature 2008, 453, 80. 18451858 10.1038/nature06932

[29] Y. Dovzhenko , F. Casola , S. Schlotter , T. X. Zhou , F. Büttner , R. L. Walsworth , G. S. D. Beach , A. Yacoby , Nat. Commun. 2018, 9, 2712. 30006532 10.1038/s41467-018-05158-9 PMC6045603

[30] J.‐P. Tetienne , T. Hingant , L. J. Martínez , S. Rohart , A. Thiaville , L. H. Diez , K. Garcia , J.‐P. Adam , J.‐V. Kim , J.‐F. Roch , I. M. Miron , G. Gaudin , L. Vila , B. Ocker , D. Ravelosona , V. Jacques , Nat. Commun. 2015, 6, 6733. 25828294 10.1038/ncomms7733

[31] K.‐ Yosida , Phys. Rev. B 1992, 46, 1232.

[32] S. S. P. Parkin , Phys. Rev. Lett. 1991, 67, 3598. 10044776 10.1103/PhysRevLett.67.3598

[33] J. C. Slater , J. Chem. Phys. 1964, 41, 3199.

[34] C. He , G. Yu , C. Grezes , J. Feng , Z. Zhao , S. A. Razavi , Q. Shao , A. Navabi , X. Li , Q. L. He , M. Li , J. Zhang , K. L. Wong , D. Wei , G. Zhang , X. Han , P. K. Amiri , K. L. Wang , Phys. Rev. Appl. 2018, 10, 1.

[35] S. Jaiswal , K. Litzius , I. Lemesh , F. Büttner , S. Finizio , J. Raabe , M. Weigand , K. Lee , J. Langer , B. Ocker , G. Jakob , G. S. D. Beach , M. Kläui , Appl. Phys. Lett. 2017, 111, 022409.

[36] J. Torrejon , J. Kim , J. Sinha , S. Mitani , M. Hayashi , M. Yamanouchi , H. Ohno , Nat. Commun. 2014, 5, 4655. 25130480 10.1038/ncomms5655

[37] A. Belabbes , G. Bihlmayer , S. Blügel , A. Manchon , Sci. Rep. 2016, 6, 24634. 27103448 10.1038/srep24634 PMC4840381

[38] H. Yang , O. Boulle , V. Cros , A. Fert , M. Chshiev , Sci. Rep. 2018, 8, 1. 30120368 10.1038/s41598-018-30063-y PMC6097993

[39] A. Fert , N. Reyren , V. Cros , Nat. Rev. Mater. 2017, 2, 17031.

[40] M. Heide , G. Bihlmayer , S. Blügel , Phys. Rev. B 2008, 78, 140403. 10.1103/PhysRevLett.101.027201 18764220

[41] A. Kubetzka , M. Bode , O. Pietzsch , R. Wiesendanger , Phys. Rev. Lett. 2002, 88, 4. 10.1103/PhysRevLett.88.057201 11863771

[42] M. J. Donahue , D. G. Porter , “OOMMF User's Guide, Version 1.0” Interagency Report NISTIR 6376, Gaithersburg, 1999, can be found under https://math.nist.gov/oommf/, n.d.

[43] A. Bellec , S. Rohart , M. Labrune , J. Miltat , A. Thiaville , Europhys. Lett. 2010, 91, 17009.

[44] M. J. Meijer , J. Lucassen , F. Kloodt‐Twesten , R. Frömter , O. Kurnosikov , R. A. Duine , H. J. M. Swagten , B. Koopmans , R. Lavrijsen , F. Kloodt‐Twesten , R. Frömter , R. A. Duine , Phys. Rev. Lett. 2020, 124, 207203. 32501071 10.1103/PhysRevLett.124.207203

[45] J. J. Yang , D. B. Strukov , D. R. Stewart , Nat. Nanotechnol. 2013, 8, 13. 23269430 10.1038/nnano.2012.240

[46] D. Kuzum , R. G. D. Jeyasingh , B. Lee , H. S. P. Wong , Nano Lett. 2012, 12, 2179. 21668029 10.1021/nl201040y

[47] E. Covi , S. Brivio , A. Serb , T. Prodromakis , M. Fanciulli , S. Spiga , Front. Neurosci. 2016, 10, 1. 27826226 10.3389/fnins.2016.00482 PMC5078263

[48] V. Milo , G. Pedretti , R. Carboni , A. Calderoni , N. Ramaswamy , S. Ambrogio , D. Ielmini , in 2016 IEEE Int. Electron Devices Meet., IEEE, Piscataway, NJ 2016, pp. 16.8.1–16.8.4.

[49] C. Burrowes , A. P. Mihai , D. Ravelosona , J.‐V. Kim , C. Chappert , L. Vila , A. Marty , Y. Samson , F. Garcia‐Sanchez , L. D. Buda‐Prejbeanu , I. Tudosa , E. E. Fullerton , J.‐P. Attané , Nat. Phys. 2010, 6, 17.

[50] M. Wu , M. Hong , C. Chang , P. Sahu , J. Wei , A. Ann , H.‐Y. Lee , S.‐S. Shcu , T.‐H. Hou , in 2019 Symp. VLSI Technol., IEEE, 2019, pp. T34–T35.

[51] A. Sengupta , A. Banerjee , K. Roy , Phys. Rev. Appl. 2016, 6, 064003.

