# Supplementary material for: Spin‐Torque Memristors Based on Perpendicular Magnetic Tunnel Junctions for Neuromorphic Computing
Source: Adv Sci (Weinh). 2021 Mar 8;8(10):2004645. doi: 10.1002/advs.202004645 (PMC8132064; doi:10.1002/advs.202004645)
Supplement: Supplementary file 1 — Supporting Information [file ADVS-8-2004645-s002.pdf]

© 2021 Wiley-VCH GmbH

ADVANCED SCIENCE
Open Access

Supporting Information

for *Adv. Sci.,* DOI: 10.1002/advs.202004645

Spin-torque memristors based on perpendicular magnetic tunnel junctions for neuromorphic computing

*Xueying Zhang, Wenlong Cai, Mengxing Wang, Biao Pan, Kaihua Cao, Maosen Guo, Tianrui Zhang, Houyi Cheng, Shaoxin Li, Daoqian Zhu, Lin Wang, Fazhan Shi, Jiangfeng Du and Weisheng Zhao***

Supporting Information

Spin-torque memristors based on perpendicular magnetic tunnel junctions for neuromorphic computing

*Xueying Zhang, Wenlong Cai, Mengxing Wang, Biao Pan, Kaihua Cao , Maosen Guo, Tianrui Zhang, Houyi Cheng, Shaoxin Li, Daoqian Zhu, Lin Wang, Fazhan Shi, Jiangfeng Du and Weisheng Zhao**

Section 1. More information about the multilayer stack

The MTJ films were deposited by a Singulus TIMARIS 200 mm magnetron sputtering machine at a base pressure of 3.75×10-9 Torr.

S1.1 Saturation magnetization and perpendicular anisotropy energy

Both the in-plane and out-of-plane (perpendicular) hysteresis loops of the film used to fabricate the magnetic tunnel junction (MTJ) were measured via vibrating sample magnetometry (VSM), as shown in **Figure S1**a. Both the free layer (FL) structure and the reference layer exhibited good perpendicular magnetic anisotropy (PMA). Furthermore, the dual FLs were globally well ferromagnetically coupled.

After producing a film (FL-film) with the same structure as the FL structure of the MTJ stack, we characterized the in-plane (Figure S1b) and out-of-plane (Figure 4a in the main text) hysteresis loops. The saturation magnetization M_S of the FLs in the MTJ was estimated based on the perpendicular hysteresis loop of the FL film. M_S was estimated to be approximately 1.0×10^6 A/m at room temperature.

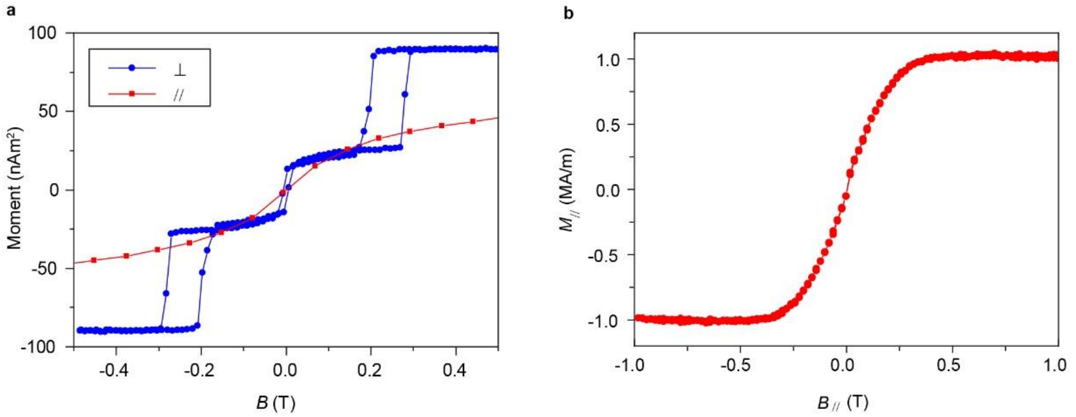

Figure S1. Hysteresis loop of magnetic film studied. a) Perpendicular and in-plane hysteresis loops of the multilayer stack used to fabricate the MTJ, measured via VSM at room temperature. b) In-plane hysteresis loop of the FL film measured via VSM at room temperature.

The perpendicular anisotropy constant K_U of the FLs was estimated based on the in-plane field hysteresis loop: the critical field that rotates the magnetization of the FLs to the in-plane direction is considered as the effective anisotropy field $\mu_0 H_{K,eff}$. The uniaxial anisotropy energy was calculated as follows:[1] $K_U = \frac{1}{2}\mu_0 H_{K,eff} M_S + \frac{1}{2}\mu_0 M_S^2$. At room temperature, the effective anisotropy field $\mu_0 H_{K,eff}$ of the FL was measured to be 250 mT, and the anisotropy KU was estimated to be 7.5×105 J/m3.

S1.2 Characterization of the FL-film via NV center in diamond

Stray fields above the FL-film is scanned using NV center in diamond to check the homogeneity of the magnetization of the film. The NV center is created by implantation of 30 keV N_2^+ ions into [100] bulk diamond and annealing for 2 hours at 1000 °C in vacuum. Therefore, the depth of the NV center is estimated to be 20.7 nm.

As a comparison, we numerically calculate the fluctuation of the stray field above a sample caused by an eventual defect, where the magnetization is locally changed. We suppose a magnetic film with homogenous magnetization $M_S = 1.0 \times 10^6$ A/m, except for one 10 nm×10 nm defect in the center where the magnetization reduces by 10%, as shown in Fig S2a. The thickness of the film is 1.8 nm and the magnetization is saturated in the z-direction. The NV center is placed 20 nm above the sample plane and the NV quantization axis is in the X-Z plane, tilting 35° from the sample plane. Micromagnetic simulation software MuMax3[2] is used to calculate the stray field projected along the NV quantization axis in the detection plane. The area considered in the simulation is 150 nm×150 nm, with 20×20 periodic repetitions to minimize the effect of boundary conditions. The simulation result (**Figure S2**b) shows that a fluctuation of stray field caused by such an isolated defect is in the order of 300 μT. Whereas,

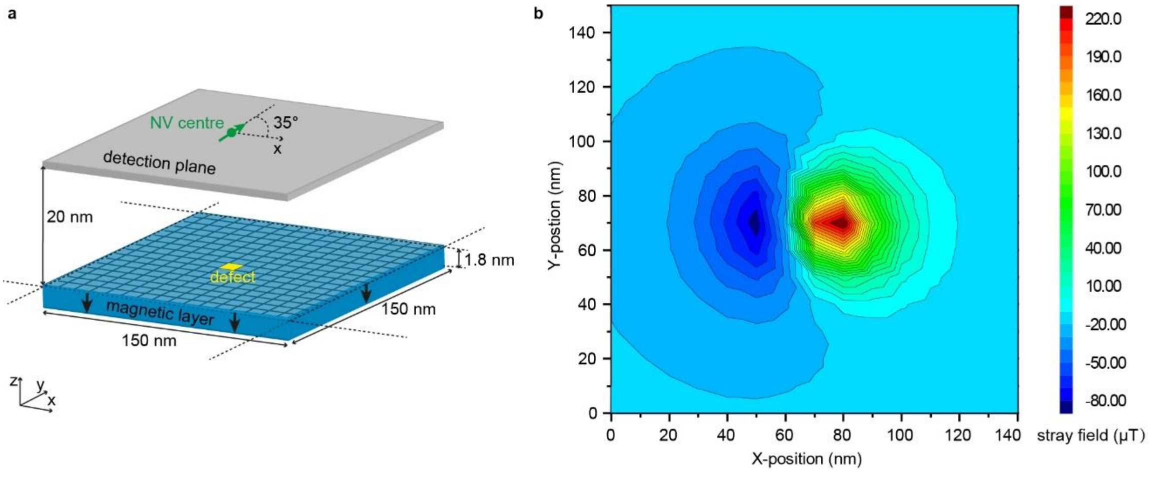

Figure S2 Numerical evaluation of the stray field detected by the NV centre. a) Schematic diagram of simulation configuration. b) Distribution of the stray field in the detection plane, projecting along the NV quantization axis.

the experimentally detected stray fields via the NV center are in the order of 80 μT. This comparison proves that the magnetization of the FL of the studied film is very homogeneous.

It should be noticed that the bias of stray field shown in Figure S2b is caused by the finite dimension of the simulated area, which is much smaller than the experimentally measured sample.

S1.3 Dzyaloshinskii-Moriya interactions (DMIs) in films with different symmetry breaking

We have measured the DMI in two films with different symmetry breaking, i.e., a MgO/CoFeB(1.7 nm)/W film and a W/CoFeB(1 nm)/MgO film. At the same time, the overall DMI in the MgO/CoFeB/W/CoFeB/MgO dual FLs film is also measured. All films are annealed at the same temperature. Measurements are based on the asymmetric domain wall (DW) motion when driven by a perpendicular field under the application of a varying in-plane field.[3,4] High perpendicular fields $B_{\perp}$ are used so that the DW moves beyond the thermally activated creep

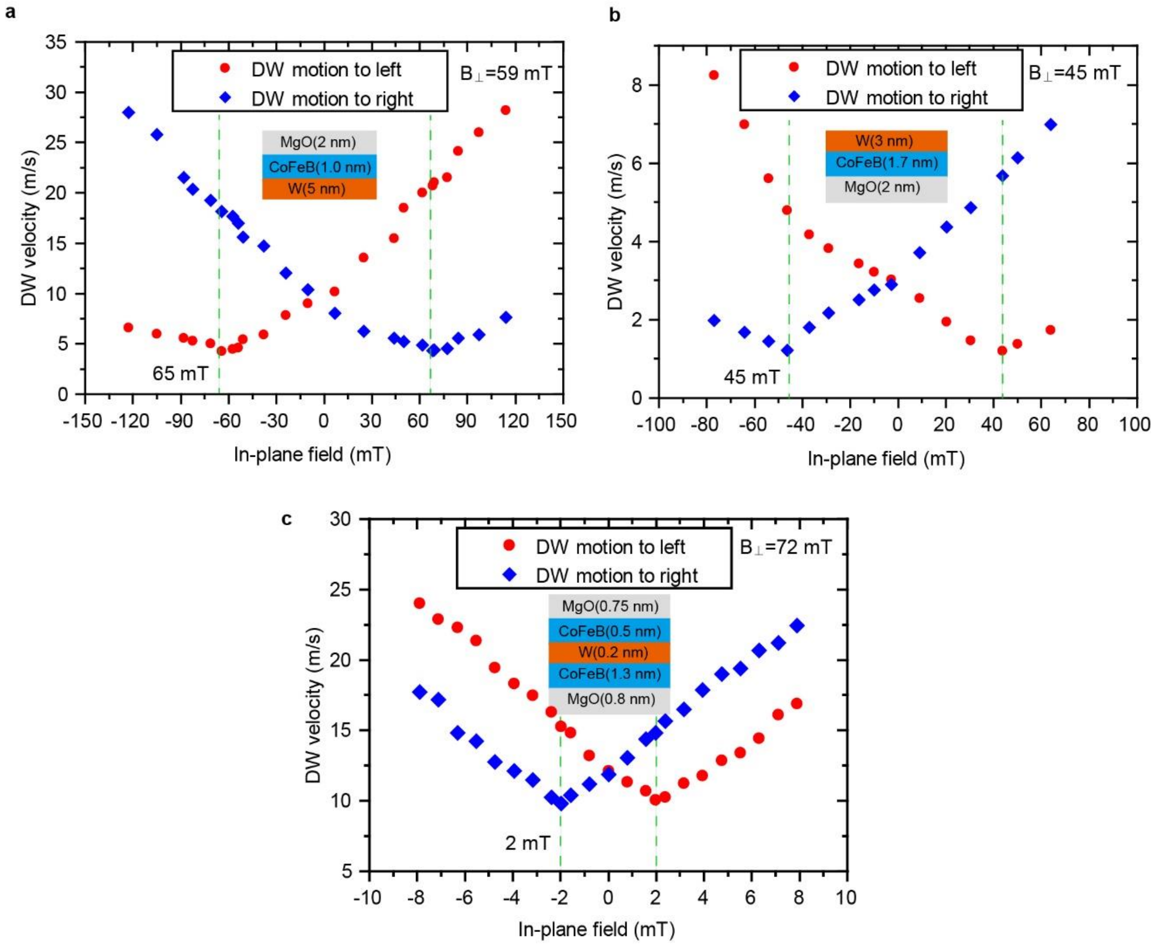

Figure S3. Measurements of the DMI in three different films structures based on asymmetrical DW motion when an in-plane field is applied. The film structure is: a) W/CoFeB/MgO; b) MgO/CoFeB/W; c) MgO/CoFeB/W/CoFeB/MgO. The magnitude of perpendicular field $\boldsymbol{B}_{\perp}$ used is given in upper right of each figure.

regime. Pinning effects enhanced by the application of in-plane fields[5] have no influence in this regime and the measured DMI is believed to be more accurate.[4] The results are shown in **Figure S3**. In each measurement, the perpendicular remains unchanged while the in-plane field varies step by step. The in-plane field under which the DW velocity reaches a minimum value can be regarded as the effective DMI field H_{DM}. Accordingly, the strength of the overall DMI can be estimated as $D = \mu_0 H_{DM} M_S \sqrt{A_{ex}/K_{eff}}$. Here, A_{ex} is the Heisenberg exchange stiffness and K_{eff} is the effective anisotropy constant.

It is found that the $\mu_0 H_{DM}$ in the W/CoFeB/MgO film is as large as 65 mT and the $\mu_0 H_{DM}$ in the MgO/CoFeB/W film is about 45 mT. The corresponding DMI constant is estimated to be 0.65 mJ/m^2 and 0.45 mJ/m^2. Here, M_S=1.0×10^6 A/m^2, $A_{ex} = 1.3 \times 10^{-11}$ J/m and $K_{eff} = 1.25 \times 10^5$ J/m^3 is used. While the asymmetry of DW motion shows opposite direction, suggesting that the sign of the DMI in these two films is different. On the contrary, in the MgO/CoFeB/W/CoFeB/MgO dual FLs film, although some slight asymmetry of the DW motion velocities was observed, the $\mu_0 H_{DM}$ was found to be as small as 2 mT and the estimated DMI constant was calculated to be D=0.02 mJ/m^2. This is a very weak value. This weak DMI can be explained by the symmetry of the MgO/CpoFeB/W/CoFeB/MgO structure. Although a considerable DMIs exist at each MgO/CoFeB and CoFeB/W interface, the overall DMI cancels out.

S1.4 Ruderman–Kittel–Kasuya–Yosida (RKKY) exchange through the W spacer layer

S. Parkin measured the RKKY exchange of two magnetic layers separated by a W layer.[6] The structure used was Si/Cr (3.5 nm)/[Co (1.5 nm)/W (x nm)]16/Cr (2 nm), with in-plane magnetic anisotropy in the Co layer. The following results were reported: the first antiferromagnetic coupling peak occurs at t_W=0.55 nm, and the antiferromagnetic coupling strength is 0.03 mJ/m2 at this peak. The thickness of W spacer that corresponds to this first antiferromagnetic region is approximately 0.3 nm.

We fitted these data using the well-known RKKY law:[7]

$$J(t_W) = A \cdot F(2k_F t_W) \quad \text{(S1)}$$

$$F(x) = \frac{x \cos x - \sin x}{x^4} \quad \text{(S2)}$$

where A is a parameter related to the magnitude of the coupling strength and k_F is a parameter related to the oscillation period. The fitting results are shown in **Figure S4**. From this fit, we

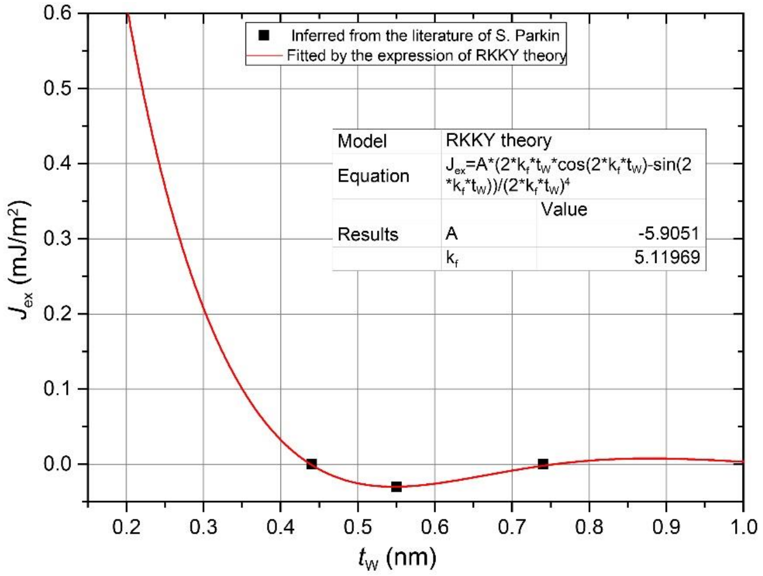

Figure S4. Strength of the RKKY coupling Jex of two ferromagnet (FM) layers as a function of the thickness of an intervening W spacer layer. The black squares represent the data reported by S. Parkin [Phys. Rev. Lett. 67, 3598 (1991)], and the red line represents the fitting results based on the RKKY theory.

can conclude that the ferromagnetic coupling between the two magnetic layers is J_{ex}=0.6 mJ/m^2 at t_W=0.2 nm and drops to zero at t_W=0.43 nm.

Section 2. Calculation of the domain wall surface energy

In this calculation, we suppose that the two FLs are mirror-symmetric with respect to the spacer layer. The tilt angle of the magnetization in the center of a DW ($\vec{m}_{\mathrm{DW}}$) with respect to the transverse direction is θ (−θ) in the LwFL (UpFL), where $\theta \in [0, \pi/2]$. The total magnetic energy of the DW is[7,8]

$$\sigma_{DW} = \sigma_0 + J_{ex}(1 - \cos 2\theta)\,\Delta/t_M - 2\pi D \sin\theta + \sigma_d \tag{S3}$$

where $\sigma_0 = 4\sqrt{A_{ex}K_{eff}}$ is the surface energy of a Bloch DW with θ=0,[9] as described in the first case in Figure 5b, but without considering the DMI energy, A_{ex} is the exchange stiffness, K_{eff} is the effective anisotropy field, Δ is the DW width, t_M is the thickness of a single FL, and σ_d is the energy associated to the dipole-dipole interaction.

The local dipole-dipole interactions between the DWs in the two FLs promote the formation of a chiral vortex DW because this type of DW configuration has a lower magnetic static energy than a coupled Bloch DW.[10] Since the theoretical calculation of this energy is very difficult, here, we perform a numerical calculation using MuMax3.[2]

The simulated structure is shown in **Figure S5**. The thicknesses of the lower FL (LwFL), the W spacer and the upper FL (UpFL) were 1 nm, 0.2 nm and 1 nm, respectively. The area of the simulated zone was 32 nm×16 nm (In fact, 30 nm is the typical geometrical width of a DW

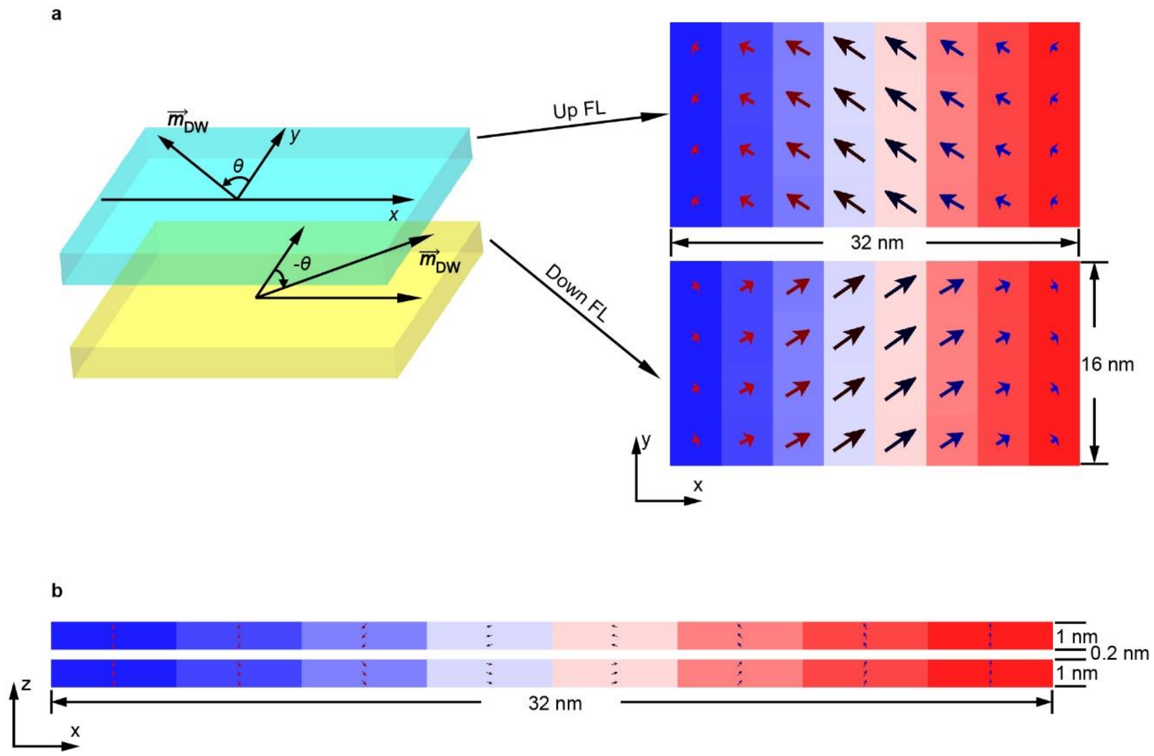

Figure S5. Estimation of the demagnetizing energy by means of a micromagnetic simulation. a) Left: sketch of the simulated structure. Right: one frame showing the magnetizations of the UpFL and LwFL viewed from the z direction during the simulation. b) One frame showing the magnetic texture viewed from the y direction during the simulation.

in a film with PMA[10]). The saturation magnetizations of the two magnetic layers were both 1.0×10^{6} A/m, and the perpendicular anisotropy energy was 7.5×10^{5} J/m^3. The exchange stiffness in each layer was $A_{ex}=1.3\times10^{-11}$ J/m. A coupled Bloch DW configuration was created as the initial state. Then, the DW center magnetization $\vec{m}_{DW}$ was tuned by means of a locally applied external field. The angle of $\vec{m}_{DW}$ in the LwFL (UpFL) θ with respect to the y-axis was varied from 0 to π/2 (0 to -π/2). The total demagnetizing energy for the simulated system, E_d, was saved during the simulation.

Then, the DW energy per unit area associated with the dipole-dipole interactions was calculated as follows:

$$\sigma_d = \frac{E_d - E_{d0}}{t_m w} \tag{S4}$$

where E_{d0} is the demagnetizing energy of the whole simulated system when θ=π/2, which is taken as the reference level of the demagnetizing energy; t_m=2 nm is the total magnetic layer thickness; and w=16 nm is the width of the simulated structure, i.e., the length of the DW.

The variation in σ_d as a function of θ is plotted in **Figure S6**, which can be fitted by $\sigma_d \approx A(1+\cos 2\theta)$. For this simulated configuration, the DW energy associated with the dipole-

dipole interactions decreases by more than 1.6 mJ per unit area when the DW structure changes from the coupled Bloch configuration to the chiral vortex configuration. This is an important factor for the formation of chiral vortices.

After substituting σ_d with $A(1 + \cos 2\theta)$ in Eqution S3, we get,

$$\sigma_{DW} = \sigma_0 + J_{ex}(1 - \cos 2\theta)\,\Delta/t_M - 2\pi D \sin\theta + A(1 + \cos 2\theta) \tag{S5}$$

where A≈0.78 mJ/m2 in our case. By minimizing the DW surface energy using $\frac{\partial \sigma_{DW}}{\partial \theta} = 0$, we obtain the value of θ at equilibrium:

$$\theta \approx \begin{cases} \arcsin \frac{\pi D t_M}{2J_{ex}\Delta - 2A * t_M} & for \left|\frac{\pi D t_M}{2J_{ex}\Delta - 2A * t_M}\right| < 1 \\ \frac{\pi}{2} & otherwise \end{cases} \tag{S6}$$

For $K_{\text{eff}} = 1.25 \times 10^5 J/m^3$, as measured via vibrating sample magnetometry (VSM) for the FLs, $A_{\text{ex}} = 1.3 \times 10^{-11}$J/m, [11] Δ=5 nm, [8] t_M=1 nm, and D=±0.5 mJ/m2, the variations of θ and the DW surface energy as functions of the interlayer coupling strength J_{ex} is obtained, as plotted in Figure 5c in the main text.

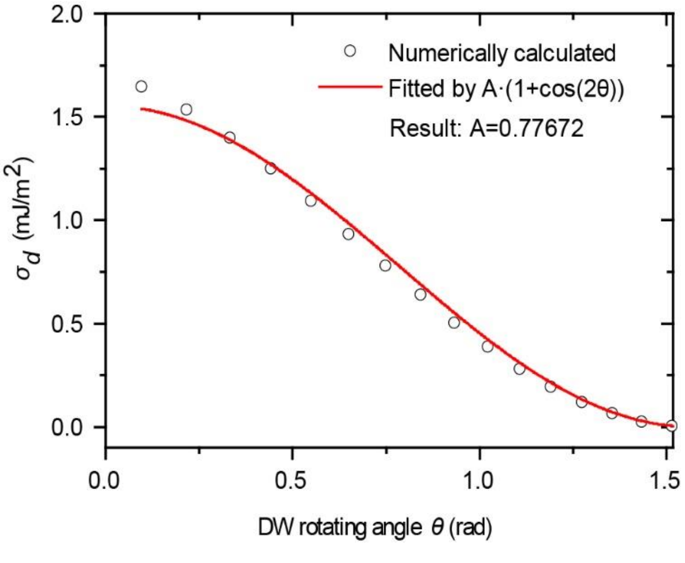

Figure S6. Variation of the DW energy in the two FLs contributed by dipole-dipole interactions as a function of the DW rotating angle *θ*, which is extracted from micromagnetic simulations. The red line is the fitting results.

Section 3. Effect of the demagnetizing field on the stability of an intermediate state

The global demagnetizing energy of an FL is reduced if the FL is partially switched because the net magnetization approaches zero. In other words, a demagnetizing field B_{demag} helps to stabilize a DW in an FL disc. Here, we use the concept of the effective magnetization current to evaluate the influence of such a demagnetizing field on the DW dynamics in the FL.[12,13] For

a uniformly magnetized film with perpendicular anisotropy, the demagnetizing field (or stray field) is equivalent to the Oersted field produced by an effective current loop around the edge of the film. The amplitude of this current is $I=M_S t_M$, where M_S is the saturation magnetization and t_M is the thickness of the magnet. Here, we suppose that the magnetic state of a partially switched FL is shown in **Figure S7**a. The DW is assumed to be a straight vertical line for simplicity of calculation, and the horizontal coordinate of the DW is x_{DW}. The B_{demag} from the FL that is acting on the DW (at point P) is equal to the Oersted field from a current loop along the edge of the disc with the current direction shown in Figure S7a. For $M_S=1\times10^6$ A/m, we numerically calculated the B_{demag} acting on the DW as a function of the horizontal coordinate x_{DW}, and the results are shown in Figure S7b. As seen from these results, the B_{demag} always favors the stabilization of the DW in the center of the disc, and its magnitude increases as the DW approaches the edge. Moreover, the magnitude of B_{demag} increases proportionally with the thickness of the magnetic layer. In our MTJ with a 1.8-nm-thick FL and a 200-nm radius, B_{demag}

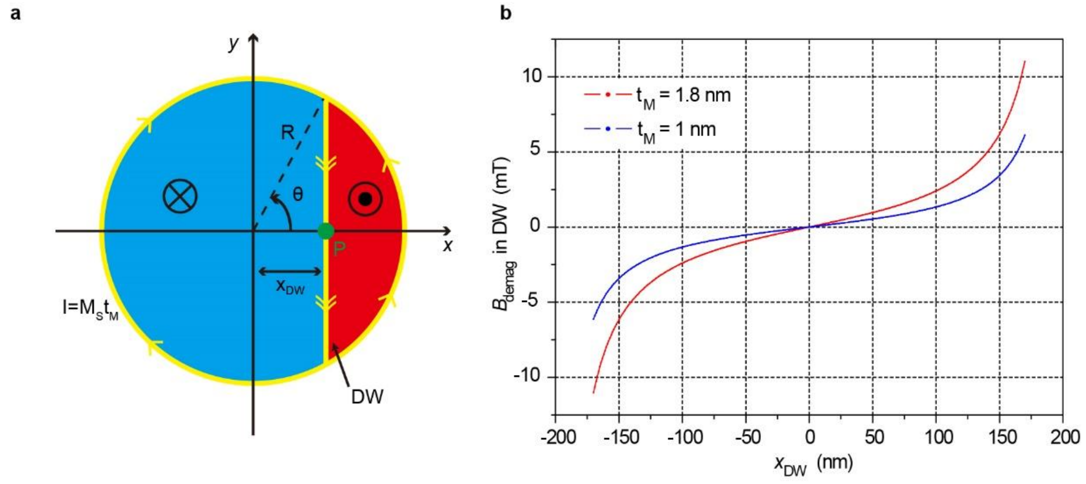

Figure S7. Calculation of the demagnetizing field B_{demag} produced by an FL disc and acting on a DW in this disc. a) Sketch showing the configuration assumed for the calculation and the effective magnetization current (yellow line). b) The demagnetizing field acting on the DW as a function of the DW position x_{DW} in the FL disk for different FL thicknesses t_M.

increases to approximately 10 mT at the edge; thus, it plays a non-negligible role in stabilizing the intermediate state before complete switching is achieved.

Section 4. DW pinning effect induced by the chiral vortex

We have simulated the pinning effect induced by the chiral vortex formed under the antiferromagnetic coupling of the two FLs and the opposing DMIs via OOMMF code. The simulated area, view from the +z direction, was 40 nm×60 nm. The film consists of three layers, i.e., the LwFL(ferromagnetic, 1 nm)/Spacer (nonferromagnetic, 0.2 nm)/UpFL(ferromagnetic, 1 nm). The two FLs were ferromagnetically coupled because of RRKY interactions, with a

coupling strength J_{ex}=0.6 mJ/m^2. However, the two FLs lost the interlayer coupling in an 8-nm wide area in the middle of the film. Parameters for the simulations were: M_S=1×10^6 A/m, K_u=7.5×10^5 J/m^3, A_{ex}=1.3×10^{-11} J/m for each FLs. DMIs with a positive (negative) sign was set in the LwFL (UpFL), |D|=0.5 mJ/m^2.

In the beginning, a DW was set at the left of the film as the initial state. Then a 10 mT perpendicular field was applied permanently. We observed that the DW moved to the right and then entered the area where RKKY interactions lost. At the same time, the structure of the DW changes from Bloch type to chiral vortex. As calculated before, the chiral vortex wall can minimize both the DMI energy and demagnetizing energy. Therefore, this structure served as an energy well and robustly pinned the DW. No further DW motion occurred in the following. The magnetic state of the film was saved during the simulation, and two frames of them can be found in **Figure S8** and the video can be found in the Supplementary material Movie S1 (separate file).

a

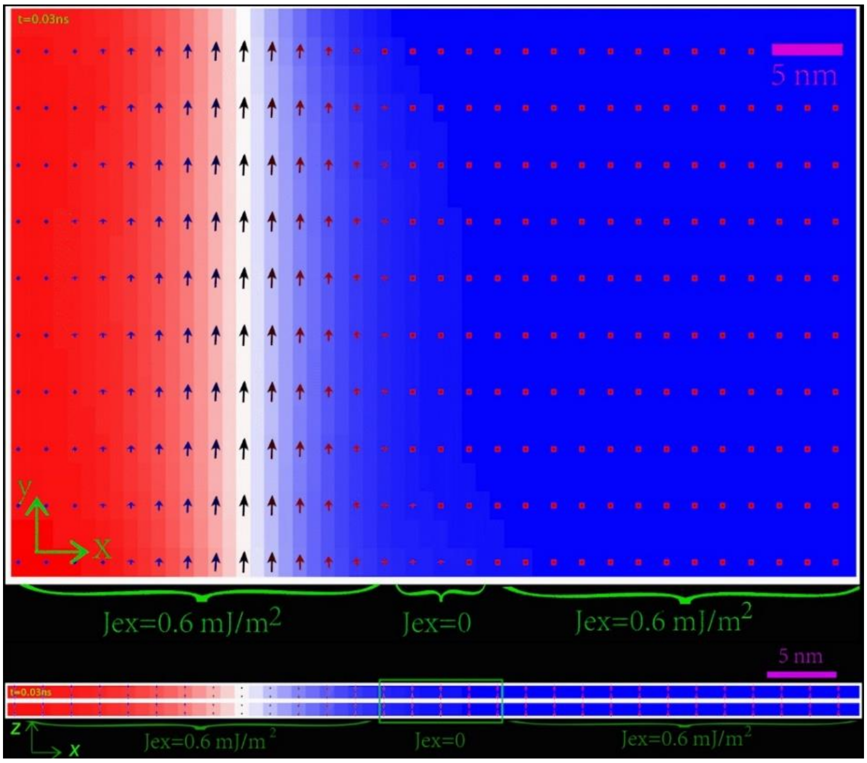

b

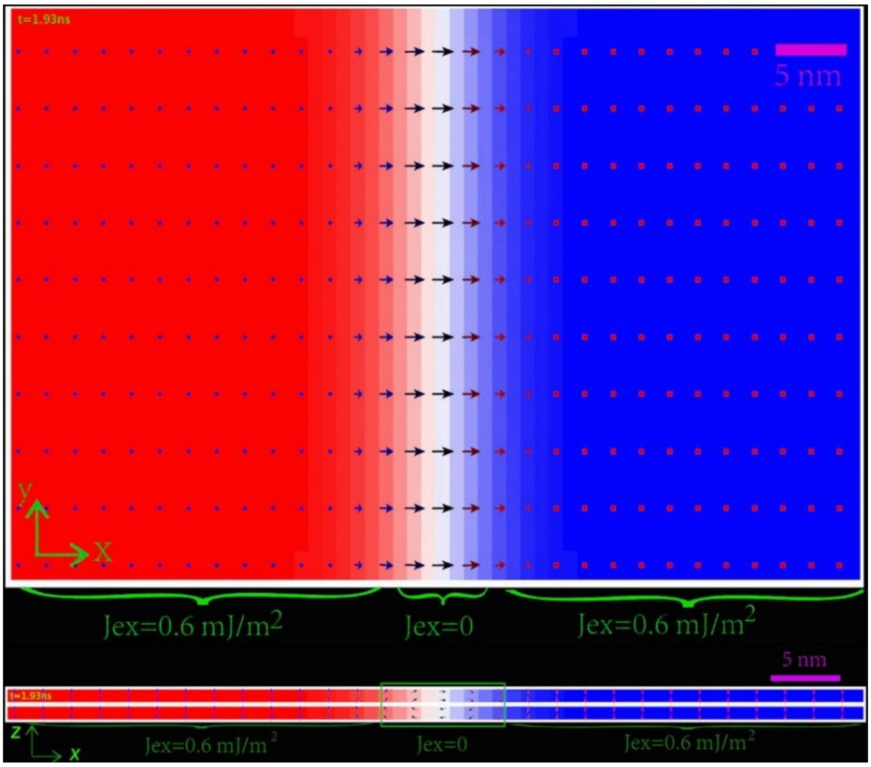

Figure S8. Two frames of video showing the DW pinning effect around the chiral vortex. (A) DW structure in area where the UpFL and LwFL is ferromagnetically coupled. (B) DW structure after entering an area where the coupling of the two FLs drops to zero. The video can be found in the supplementary material Movie S1 (separate file). The figure in the up of each figure is the top view and figure in the lower is the side view.

Movie S1 (separate file). Movie showing the structural transformation of a DW when it moves from a region with strong interlayer coupling to a region with weak interlayer coupling, and the DW pinning effect induced by the formation of the chiral vortex. Detailed simulation parameters are given in section S4 of supplementary material S1.

Section 5. Model for the memristive behavior

For both the P to AP and AP to P switching process in our device, the variation of the resistance with time is difficult to be accurately described with a theoretical model. However, it can be phenomenologically fitted by an exponential function, with a characteristic time τ, as shown in Figure 6a in the main text. $\ln\tau$ is found to be linear to the magnitude of applied voltage, as shown in **Figure S9**. In detail, for the P to AP switching, the change of device resistance with time under a certain voltage V can be described as,

$$R(t) = R_{AP} - (R_{AP} - R_P) \cdot e^{t/\tau^+} \quad \text{(S7)}$$

where $\ln\tau^+ = a^+ + b^+V$. Here, we defined the voltage inducing P to AP switching as positive, and τ^+ represents the characteristic time for P to AP switching. Similarly, for AP to P switching,

$$R(t) = R_P + (R_{AP} - R_P) \cdot e^{t/\tau^-} \quad \text{(S8)}$$

where $\ln\tau^- = a^- + b^-V$, and a^+, b^+, a^-, b^- are constant. Based on a set of experimental measurements and statistical analyses, we get $a^+ = 142.28$, $b^+ = -283.97$, $a^- = 48.28$, $b^- = 135.8$, where the unit of τ is s and V is V.

Given a device with a resistance of R_j at the present state, when a spike arrives (a voltage pulse with amplitude V and duration T_P), the resistance change δR depends on the R_j, V, T_P and the polarity of the pulse,

$$\delta R = \frac{1}{\frac{\partial t}{\partial R}|_{R=R_j}} T_P \quad \text{(S9)}$$

According to Equation S7 and Equation S8, $\frac{\partial t}{\partial R} = \frac{1}{R_{AP}-R} e^{a^+ + b^+V}$ for a positive pulse, and $\frac{\partial t}{\partial R} = \frac{1}{R_P - R} e^{a^- + b^-V}$ for a negative pulse. So, the resistance after the spike is

$$R_{j+1} = \begin{cases} R_j + \frac{R_{AP}-R_j}{e^{a^+ + b^+V}} T_P & for \quad V > 0 \\ R_j + \frac{R_P - R_j}{e^{a^- + b^-V}} T_P & for \quad V < 0 \end{cases} \quad \text{(S10)}$$

Furthermore, considering the stochastic variation of the resistance, which is caused by stochastic depinning of domain walls, a noise factor n_j is multiplied on the δR.

$$n_j = \frac{x_j}{2} + \frac{abs(x_j)}{2} \tag{S11}$$

where x_j is a random number with a normal distribution generated for the jth operation. Note that if x_j is positive, multiplicative noise n_j is also positive and the resistance R changes with a

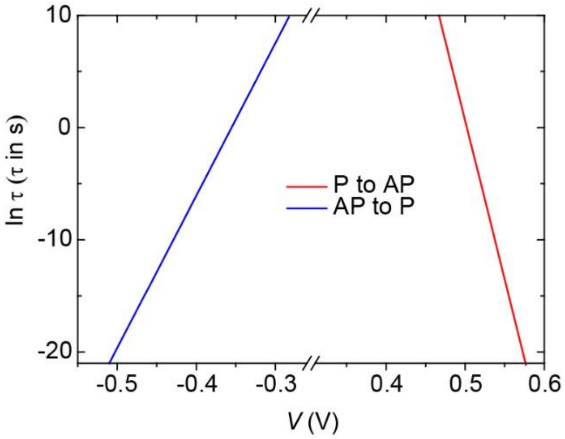

Figure S9. The relationship between the characteristic time constant τ and the applied voltage for the P to AP and P to AP switching.

finite amplitude; if x_j is negative, n_j equals zero and R does not change after the operation, corresponding to a domain wall pinning event. By carefully adjusting the mean value and standard deviation of x_j, the stochasticity of resistance change caused by devices' variation or domain wall pinning events can be adequately mimicked in the simulation. Figure 6a & b in the main text give examples of the memristive switching process obtained using the above model for fixed voltage. It is to note that we found it difficult to obtain a complex switched state under relatively low voltage during the AP to P switching process in our devices. This phenomenon is considered in the simulation and the value of R_P in Equation S10 used in the simulation is set to be 115 Ω.

Section 6. Spike neural network simulations

In this section, we will give a detailed description of SNN simulation based on the spin-torque memristor device, which could be divided into the following four steps.

The first step is the pretreatment of the input images using the receptive field function to make the input data closer to what a retina neuron would perceive.[14] As can be seen from **FigureS10**, where the input is a 20×20-pixel black-and-white image, we constructed an on-centered 5×5 receptive field array in which stimulation leads to response of a particular sensory

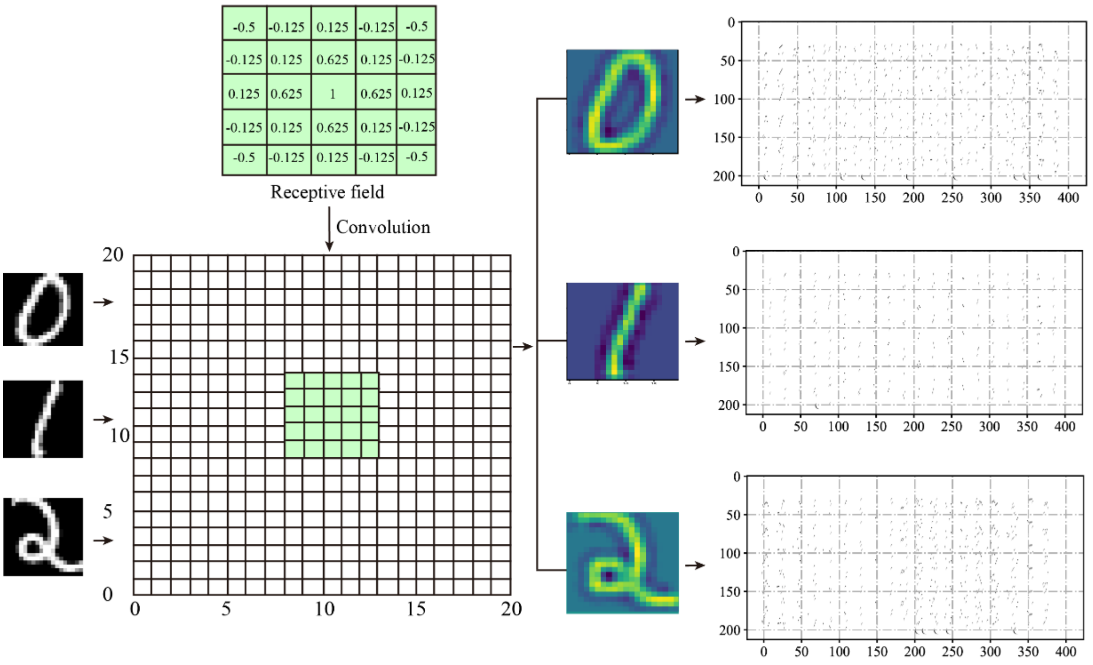

Figure S10. Illustration of the transition process from the input images to spike trains. Firstly, the original images representing three hand-written numbers went through a convolution operation by the receptive field array and be converted into the images more suitable for human eye perception. Each box of the 20×20 grid is corresponding to one pixel in the input image. The receptive field array will act as a convolution kernel sliding from beginning to the end of the grid. Then, the post-treatment images were fed to an encoder, which could convert them into spike stimulus for input neurons as shown in the right three scatter diagrams. The x-axe is the neuron number while y-axe is the spike time. Every point in the graph represent a spike.

neuron. The numbers of on-centered receptive field array are weighted according to the Manhattan Distance[15] from the center of the window. Then, a 2D convolution operation was conducted and output a color image with analog values. The second step is to convert the color images into spike trains. Image data calculated from convolution operation is an analog value and can’t be fed into the input neuron layer directly. Since the neuron’s membrane potential could only be modulated by the spike stimulus, we employ a spike-train encoder built by *interpolation function* as an interface between image data from the physical world and SNNs. The type of encoding adopted here is rate coding, which suggests that the information is carried by the firing rate of the neuron. Hence, spike trains generated with an average firing rate lies between 1-200 Hz corresponding to the input images have been shown in Figure S10.

The third step is the construction of spiking neurons. In this work, we choose a widely accepted simplified Leaky-Integrate-Fire (LIF) model as neuron research object. As illustrated in **Figure S11**a, a two-layer network composed of a set of input and output neurons were connected through synapses. Along with the arrival of encoded image information, the input neurons generate discrete events or spikes and send them to the output neurons. The spikes from

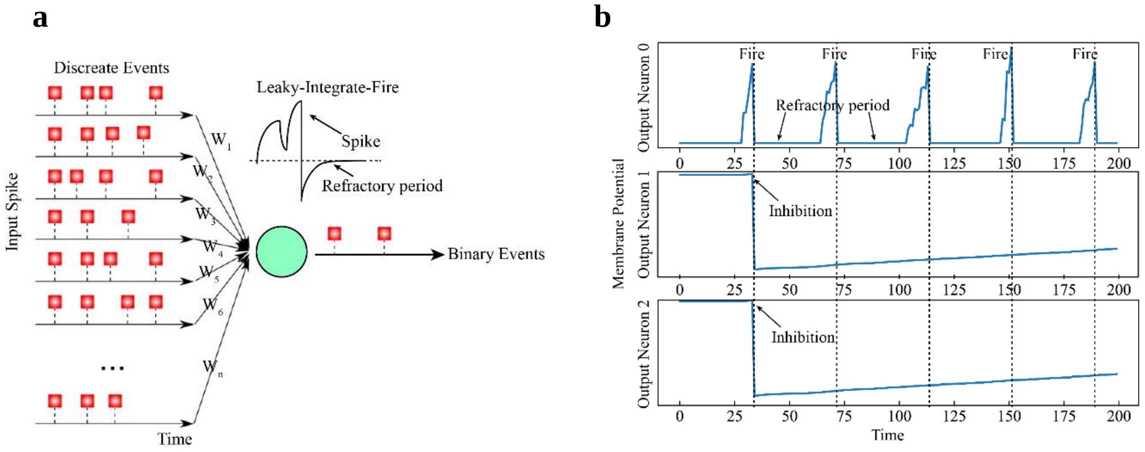

Figure S11. a) A representative model for the Leaky-Integrate-Fire (LIF) neuron used in the SNN simulation. The input spikes generated by input neurons are modulated by the synaptic weights W1~n and summed up together. The summation output alters the membrane potential of the post-neuron. The neuron will emit a spike if the membrane potential crosses a certain threshold. b) Simulation results for the 3 output neurons. Top panel shows the membrane potential of the winner neuron, exhibiting the typical LIF dynamics. No more spikes are generated during the refractory period until the device is reset to its initial potential. Middle and bottom panel shows the membrane potential of the other two output neurons that were inhibited by the fire of winner neuron.

all the pre-neurons are altered as per the associated weights $W_{1\sim n}$, and summed up as shown in Figure S11a. The output after summation alters the membrane potential (V_{mem}) of the post-neuron in a typical Leaky-Integrate-Fire fashion.[16] When the potential crosses a threshold value (V_{th}), the post-neuron emits a spike and then enters into a refractory period during which no new input is received and the V_{mem} remains constant. Meanwhile, the first firing output neuron performs lateral inhibition on the rest of neurons. Figure S11b gives an example of simulation results to verify the correctness of our model design.

The fourth step is mapping the spin-torque memristor device into synapses. The key is to determine the resistance change δR of synapses after each epoch of stimulus, depending on the spike signal received by each synapse. This change should be consistent with the characteristic of the real devices, including its stochastic nature. The model introduced in Section S5 is used. In this model, parameters of device such as R_P, R_{AP}, a^+, b^+, a^-, b^- is extracted from the experimentally measured results on real devices. Then δR depends only on three parameters, i.e., the present resistance R, the amplitude V and the duration T_{P} of spike applied on the synapse, multiplied by a noise factor. As given in the main text (Figure 5 c-e), to realize the STDP function, two sequences of ramped voltage pulses are used as pre-spike and a couple of short pulses with opposite polarity are used as post-spike. Since the amplitued of these two spikes are both lower than the critical current (see fig. 2b in the paper), neither of them can

change the resitance independently. Effectively, δR depends only on the peak of the across voltage V of the two spikes, as shown in the bottom panel in Figure 5c, which is determined by the delay time Δt between the pre- and post-spike. According to the waveform used in our experiments shown in Figure 5c, the relationship between V and Δt can be expressed as,

$$V = \begin{cases} 0.00187 \times \Delta t + 0.5525 & for\ \Delta t < 0 \\ 0.00199 \times \Delta t - 0.5544 & for\ \Delta t > 0 \end{cases} \quad \text{(S -12)}$$

where the unit of V is V and Δt is μs. This relationship is imported in the SNN simulation. After combining the Equation S10, Equation S11 and Equation S12, the STDP of the memristor can be adequately described and integrated into the simulation.

With the behavior model of the spin-torque memristor, the STDP weight update rule and the SNN framework described above, we have conducted a simulation with unsupervised learning and the result is shown in Figure 6c.

Section 7. stability of the intermediate state at room temperature

The stability of intermediate state against the magnetic field at room temperature is measured and shown in **figure S12**. It can be seen that the field needed to destroy the intermediate state drops a little compared with that at low temperature (fig.2d) because of thermal fluctuation. [17]

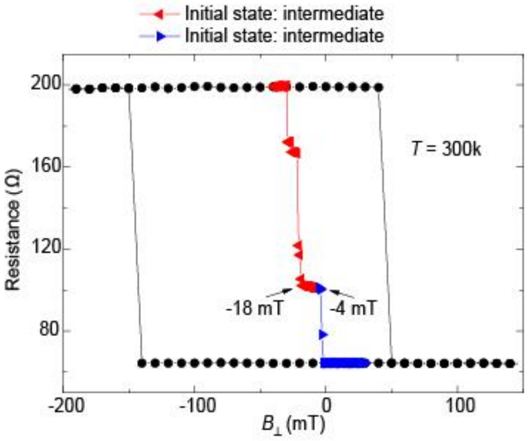

Figure S12. Stability of an intermediate state against magnetic field in the memristive MTJ at room temperature. The test method is the same as that in figure 2d in the main text.

SI References

[1] B. Zhang, A. Cao, J. Qiao, M. Tang, K. Cao, X. Zhao, S. Eimer, Z. Si, N. Lei, Z. Wang, X. Lin, Z. Zhang, M. Wu, W. Zhao, *Appl. Phys. Lett.* **2017**, *110*, DOI 10.1063/1.4973477.

[2] A. Vansteenkiste, J. Leliaert, M. Dvornik, M. Helsen, F. Garcia-Sanchez, B. Van Waeyenberge, *AIP Adv.* **2014**, *4*, 107133.

[3] S.-G. Je, D.-H. Kim, S.-C. Yoo, B.-C. Min, K.-J. Lee, S.-B. Choe, *Phys. Rev. B* **2013**, *88*, 214401.

[4] T. Koyama, Y. Nakatani, J. Ieda, D. Chiba, *Sci. Adv.* **2018**, *4*, eaav0265.

[5] M. Vaňatka, J.-C. Rojas-Sánchez, J. Vogel, M. Bonfim, M. Belmeguenai, Y. Roussigné, A. Stashkevich, A. Thiaville, S. Pizzini, *J. Phys. Condens. Matter* **2015**, *27*, 326002.

[6] S. S. P. Parkin, *Phys. Rev. Lett.* **1991**, *67*, 3598.

[7] K.- Yosida, *Phys. Rev. B* **1992**, *46*.

[8] A. Thiaville, S. Rohart, É. Jué, V. Cros, A. Fert, *EPL (Europhysics Lett.* **2012**, *100*, 57002.

[9] A. Mougin, M. Cormier, J. P. Adam, P. J. Metaxas, J. Ferré, *Europhys. Lett.* **2007**, *78*, 57007.

[10] A. Bellec, S. Rohart, M. Labrune, J. Miltat, A. Thiaville, *EPL (Europhysics Lett.* **2010**, *91*, 17009.

[11] A. V. Khvalkovskiy, V. Cros, D. Apalkov, V. Nikitin, M. Krounbi, K. A. Zvezdin, A. Anane, J. Grollier, A. Fert, *Phys. Rev. B - Condens. Matter Mater. Phys.* **2013**, *87*, 2.

[12] E. Purcell, *Electricity and Magnetism*, McGraw-Hill Book Company, New York, **1963**.

[13] N. Vernier, J. P. Adam, S. Eimer, G. Agnus, T. Devolder, T. Hauet, B. Ocker, F. Garcia, D. Ravelosona, *Appl. Phys. Lett.* **2014**, *104*, 122404.

[14] F. E. Theunissen, S. V. David, N. C. Singh, A. Hsu, W. E. Vinje, J. L. Gallant, *Netw. Comput. Neural Syst.* **2001**, *12*, 289.

[15] "Paul E. Black, 'Manhattan distance', in Dictionary of Algorithms and Data Structures [online], Paul E. Black, ed. 11 February 2019. (accessed 2020-07-03) Available from: https://www.nist.gov/dads/HTML/manhattanDistance.html," **n.d.**

[16] L. . Abbott, *Brain Res. Bull.* **1999**, *50*, 303.

[17] J. P. Attané, D. Ravelosona, A. Marty, Y. Samson, C. Chappert, *Phys. Rev. Lett.* **2006**, *96*, 1.
